# Supplementary material for: The interactome of intact mitochondria by cross-linking mass spectrometry provides evidence for coexisting respiratory supercomplexes
Source: Mol Cell Proteomics. 2017 Dec 8;17(2):216–32. doi: 10.1074/mcp.RA117.000470 (PMC5795388; doi:10.1074/mcp.RA117.000470)
Supplement: Supplemental Data [file supp_RA117.000470_133922_0_supp_23978_fzffwf.zip › spectra_annotation/mito_spectra_annotation/395-1-14-1-9-1.pdf]

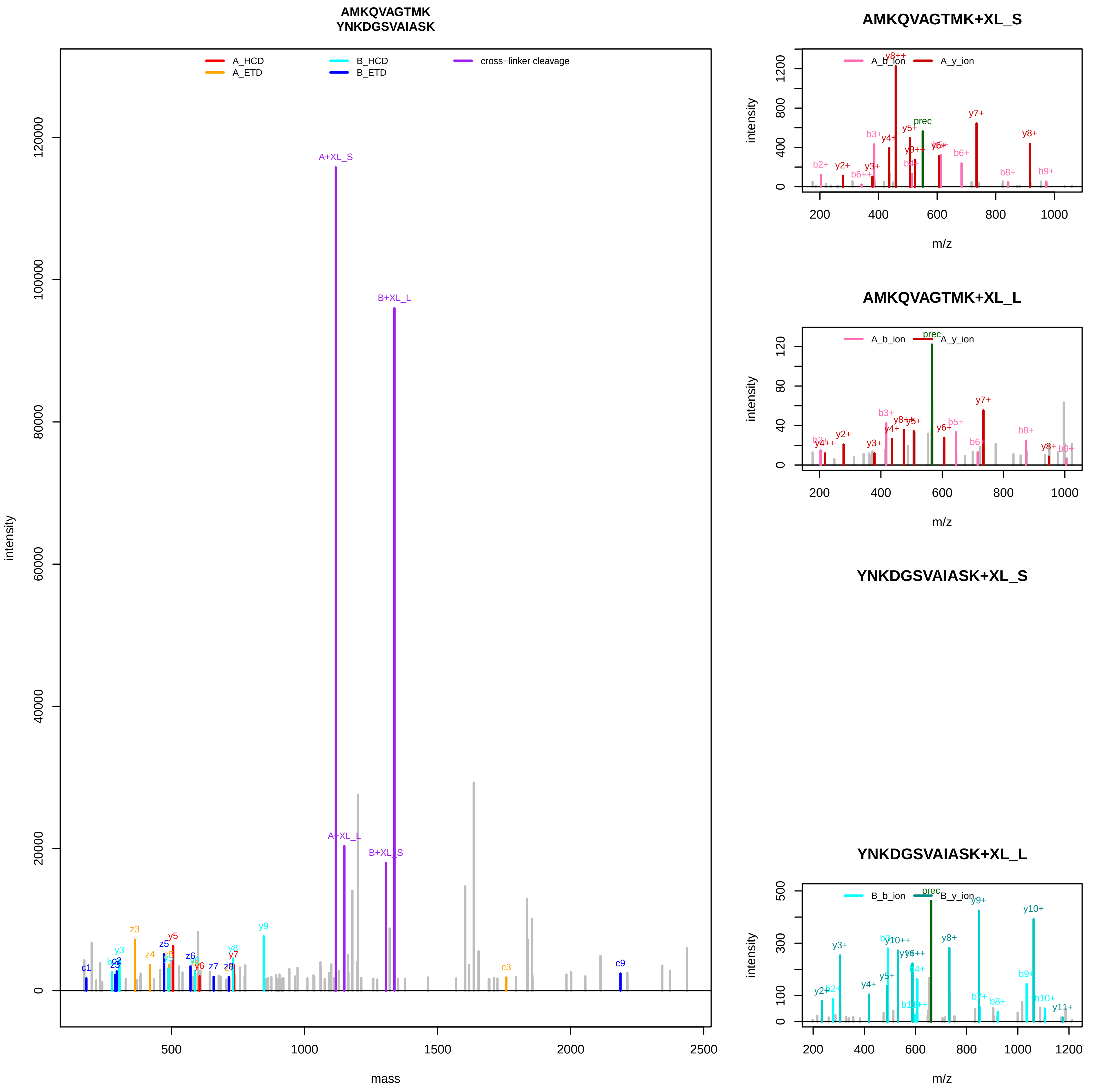
AMKQVAGTMK
YNKDGSVAIASK
A_HCD
A_ETD
B_HCD
B_ETD
cross−linker cleavage
A+XL_S
B+XL_L
A+XL_L
B+XL_S
intensity
mass
0
20000
40000
60000
80000
100000
120000
500
1000
1500
2000
2500
c1
z3
y3
z4
z5
y5
z6
y6
z7
z8
y7
y8
y9
c3
c9
AMKQVAGTMK+XL_S
A_b_ion
A_y_ion
y8++
prec
y7+
y5+
y4+
b3+
y8+
b2+
y2+
y3+
b6++
b6+
b8+
b9+
m/z
AMKQVAGTMK+XL_L
y7+
b3+
y2+
b5+
y6+
b8+
y3+
y8+
YNKDGSVAIASK+XL_S
YNKDGSVAIASK+XL_L
B_b_ion
B_y_ion
y9+
y10+
y3+
y8+
y4+
y5+
y2+
b8+
b9+
b10+
y11+
